# An Entrapped Vacuum Drainage Tube Between the Surfaces of a Dual-Mobility Cup Following Total Hip Arthroplasty

**DOI:** 10.7759/cureus.30059

**Published:** 2022-10-08

**Authors:** Kyriakos Papavasiliou, Charlie Bouthors, Victor Maigné, Charles Court

**Affiliations:** 1 Department of Orthopaedic Surgery, Kremlin-Bicêtre Teaching Hospital, Université Paris-Saclay, Le Kremlin-Bicêtre, FRA

**Keywords:** dual-mobility total hip arthroplasty, total hip arthroplasty complication, early postoperative complication, vacuum drainage tube entrapment, total hip arthroplasty

## Abstract

This paper aims to present the unique, to the best of our knowledge, case of entrapment of a standard vacuum drainage tube in the articulating surfaces of the cup of dual-mobility total hip arthroplasty. A 75-year-old woman with end-stage idiopathic avascular necrosis of the left femoral head was referred to the arthroplasty service of our tertiary orthopedic department. She underwent a scheduled and uneventful total hip arthroplasty with a press-fit dual-mobility prosthesis through a standard posterior approach. On the second postoperative day, the attempt to remove the standard vacuum drainage was unsuccessful. Consequently, the patient underwent urgent re-operation. The drain tube was found entrapped between the articulating surfaces of the posterior-inferior aspect of the dual-mobility cup and was uneventfully removed. The patient was discharged with no further events three days after her second operation. Our unique rare case increases awareness when performing even routine everyday surgical procedures because a rare complication may occur irrespective of the level of vigilance of the surgeon and can potentially compromise the outcomes of an otherwise well-performed operation.

## Introduction

Total hip arthroplasty is considered to be one of the most successful orthopedic procedures [1]. Since its introduction, subsequent and numerous improvements in the surgical techniques and the materials used have rendered it safe, efficient, and overall successful operation, accompanied by high patient satisfaction rates [1]. However, every surgical procedure, irrespective of how well-designed and performed, may be accompanied by complications [2].

We present the unique, to the best of our knowledge, case of postoperative entrapment of a standard vacuum drainage tube in the articulating surfaces of the cup of a dual-mobility cementless total hip arthroplasty, which necessitated a re-operation for its removal.

## Case presentation

A 75-year-old Caucasian woman with intractable pain in her left hip and subsequent inability to walk and perform everyday activities was referred by her general practitioner to the arthroplasty service of our tertiary orthopedic department. Her significant medical history consisted of type II diabetes mellitus, dyslipidemia, arterial hypertension, acromegaly, and mitral valve insufficiency under medication. Currently in complete remission, she had also undergone trans-anal resection of a colon carcinoma more than a decade ago.

**Figure 1:**
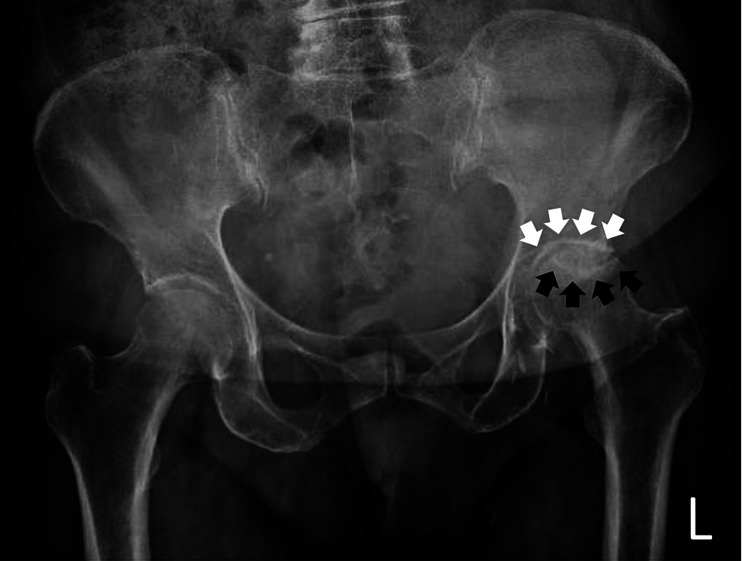
The preoperative radiograph of the patient. Preoperative standard anteroposterior hip and pelvis radiograph depicting the Ficat [3] end-stage avascular necrosis of the left femoral head. Notice the crescent sign (depicted by black arrows) and the sclerotic osteoarthritic border of the acetabulum (depicted by white arrows).

Her symptoms (hip pain, difficulty walking, and restricted hip movements) started 18 months before her referral and gradually deteriorated. Upon her initial evaluation, all her hip movements were painful and she was using two crutches for walking, being ambulatory only within her household limits. Passive hip flexion was 60°, there was a lack of extension of approximately 10°, and internal and external rotation movements were not possible. A standard radiograph revealed the existence of a Ficat [3] end-stage idiopathic avascular necrosis of the left femoral head [4] (Figure 1).

**Figure 2:**
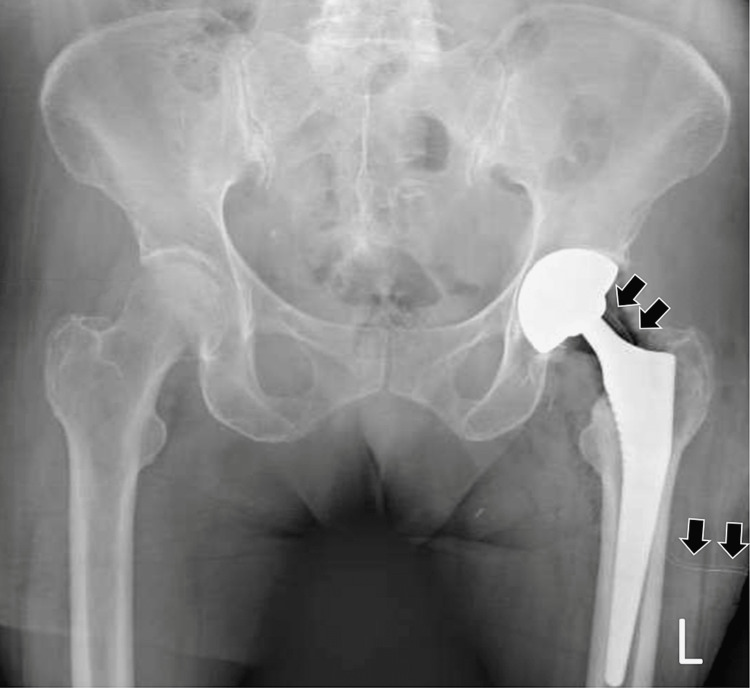
Immediate postoperative radiograph following total hip arthroplasty. Standard anteroposterior hip and pelvis radiograph taken immediately postoperatively. Notice the position of the vacuum drainage tube (as depicted by black arrows) and its course toward the posterior-inferior aspect of the dual-mobility cup.

The patient was offered a total hip arthroplasty to her left hip, which she accepted. She was operated on two months later. Under general endotracheal anesthesia and through a posterior approach, a press-fit dual-mobility total hip arthroplasty was implanted (Corin Meije Duo femoral stem paired with a Corin Dual Mobility Cup, Corin Tornier SAS, Montbonnot-Saint-Martin, France). Following the insertion of the implant and the reduction of the hip joint, and after full and unrestricted movements toward all directions were checked and secured, as a standard operative procedure, a 14 Redon vacuum drainage tube was inserted and placed near the articulation. After the completion of the operation, a standard hip and pelvis radiograph was taken (Figure 2).

**Figure 3:**
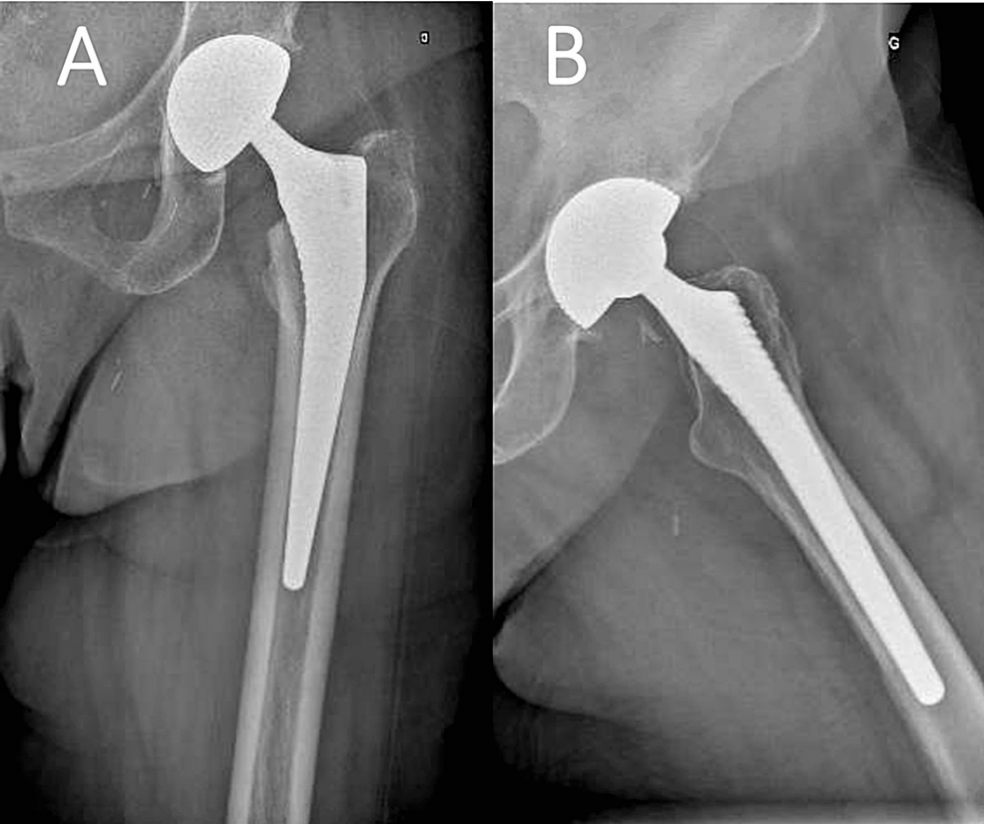
Radiograph obtained six months postoperatively. Standard anteroposterior (A) and lateral (B) radiographs of the operated hip at six months postoperatively.

On the second postoperative day, the Redon canister was filled with 160 ccs of blood and it was decided to be removed. However, the attempt to remove it was unsuccessful. Consequently, the patient underwent urgent re-operation. The drain tube was found entrapped between the articulating surfaces of the posterior-inferior aspect of the dual-mobility cup. It was removed by gently yet firmly applying extraction force while the hip was slightly flexed, adducted, and internally rotated. The removed tube was checked and was found to be compressed at a distance of 4 mm from its tip, without any sign of a rupture. No evidence of a remaining part of the tube could be found, and full and unrestricted movements of the hip joint were confirmed. The wound was thoroughly irrigated with saline and sutured in layers. The standard perioperative antibiotic protocol for patients undergoing total hip arthroplasty (three doses of 2 g of cefazolin during 24 hours) was used for the re-operation. The patient was released with no further events and on schedule three days following her second operation. Upon her latest follow-up visit six months postoperatively, clinically, she was capable of full weight-bearing, she was able to walk without any assistance and/or aids, she reported no problem, and she was fully satisfied with the result of the operation. The radiograph taken on the same day confirmed the proper positioning of the prosthesis (Figure 3).

## Discussion

Total hip arthroplasty has been a milestone in the treatment of patients with hip osteoarthritis and in orthopedic surgery in general. Since the introduction of modern total hip arthroplasty by Sir John Charnley, it has continuously evolved [1]. New materials, new pairing couples, and different surgical approaches have contributed to its success. Dual-mobility cups, in particular, have revolutionized total hip arthroplasties to a large extent by increasing their stability and reducing postoperative dislocation rates, as well as by reducing the hip range of motion restrictions, allowing patients to live a more active life [5]. Following a comprehensive discussion with the patient and based on her needs, expectations, and limitations, a press-fit dual-mobility total hip arthroplasty was proposed to her as the most suitable solution.

Every complication or malfunction of the components of total hip arthroplasty and/or a procedure related to the operative process itself, necessitating a re-operation, increases the possibility of an infection, with potentially catastrophic results [6]. This is more accurate when a re-operation needs to occur in the immediate postoperative period, as in our case, where we had to urgently re-operate on our patient only 48 hours postoperatively to remove the entrapped vacuum tube [7].

Despite thorough literature research, no data could be found regarding the incidence of vacuum drainage tube entrapment following total hip arthroplasty. This is certainly an infrequent and relatively “benign,” yet familiar to all surgeons, complication. The drainage tube is usually accidentally sutured during the closure procedure, which necessitates re-operating on the patient to be released. To the best of our knowledge, the entrapment of the drainage tube within the articulating surfaces of a dual-mobility total hip arthroplasty cup has never been reported in the literature. We postulate that this must have occurred either during the transfer of the patient from the operating table or during the immediate postoperative period and gradual mobilization because upon completion of the operation it was not evident. To prevent such complications, we recommend shortening the drain to keep it at a distance from the dual mobility to avoid entrapment during mobilization.

The limitation of our case report is the lack of a transoperative photograph documenting the entrapment of the drainage tube within the articulating surfaces of the dual-mobility cup. Nevertheless, our rare case aims to increase awareness when performing even routine everyday surgical procedures because a rare complication may occur irrespective of the level of vigilance of the surgeon and potentially compromise the outcomes of an otherwise well-performed operation.

## Conclusions

Every complication following total hip arthroplasty, which necessitates a re-operation, increases the possibility of an infection, with potentially catastrophic results. This is especially true when complications occur during the immediate postoperative period. This rare case proves that non-expected complications may occur even to experienced surgeons performing operations that they are familiar with. Extreme vigilance maintained during the whole operation and attention to every little detail (even the positioning and length of the drainage tube) are needed to secure the best outcomes.
